# Personalised prevention of type 2 diabetes

**DOI:** 10.1007/s00125-022-05774-7

**Published:** 2022-08-02

**Authors:** Nicholas J. Wareham

**Affiliations:** Medical Research Council Epidemiology Unit, Institute of Metabolic Science, University of Cambridge Clinical School, Cambridge, UK

**Keywords:** Personalised medicine, Personalised prevention, Precision medicine, Prevention, Review, Type 2 diabetes

## Abstract

It is well established from clinical trials that behavioural interventions can halve the risk of progression from prediabetes to type 2 diabetes but translating this evidence of efficacy into effective real-world interventions at scale is an ongoing challenge. A common suggestion is that future preventive interventions need to be more personalised in order to enhance effectiveness. This review evaluates the degree to which existing interventions are already personalised and outlines how greater personalisation could be achieved through better identification of those at high risk, division of type 2 diabetes into specific subgroups and, above all, more individualisation of the behavioural targets for preventive action. Approaches using more dynamic real-time data are in their scientific infancy. Although these approaches are promising they need longer-term evaluation against clinical outcomes. Whatever personalised preventive approaches for type 2 diabetes are developed in the future, they will need to be complementary to existing individual-level interventions that are being rolled out and that are demonstrably effective. They will also need to ideally synergise with, and at the very least not detract attention from, efforts to develop and implement strategies that impact on type 2 diabetes risk at the societal level.

**Graphical abstract:**

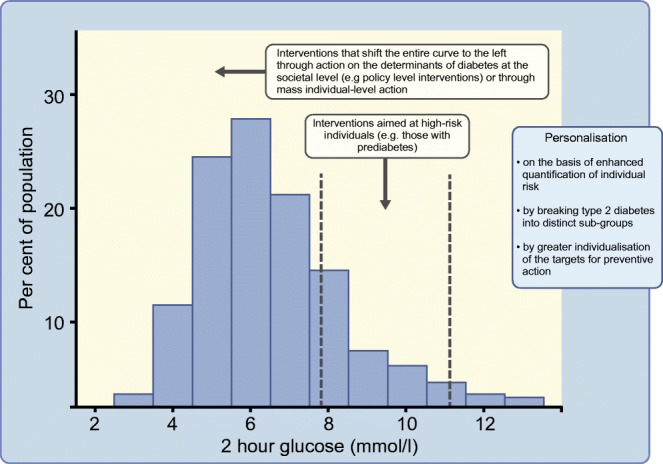

## From the establishment of efficacy to effectiveness: type 2 diabetes prevention

Twenty-five years ago, a series of RCTs began to report findings [1–6], which subsequently unequivocally demonstrated that type 2 diabetes was a preventable disease [7, 8]. This observation was crucial in focusing the attention of researchers, research funders, practitioners, policy makers and, perhaps most importantly, the wider public, on prevention. Showing that a medical condition is theoretically preventable is of course not the same as showing that the potential for prevention can be converted into real health benefit. Thus the past two decades have seen a shift in attention away from demonstrating the efficacy of preventive interventions to the implementation and evaluation of real-world strategies for prevention [8]. This has proved to be a rather more difficult challenge, not least because the design and delivery of the interventions are often beyond the sphere of influence of the researcher. In some situations, where researchers have attempted to linearly translate from diabetes prevention trials into real-world settings, there has been a marked gap between what can be achieved in the idealised context of a controlled trial and the real world [9]. Closing this gap between efficacy and effectiveness is an ongoing priority [10]. The translational challenge applies not only to the delivery of the intervention but also to the process of identification of individuals to whom it might be offered. In most of the original diabetes prevention trials people were recruited on the basis of an OGTT [2, 3]. This test helps identify individuals with impaired glucose tolerance (IGT) and, in the case of the US Diabetes Prevention Program (DPP), which coupled a trial-inclusion criterion of IGT (defined by 2 h post-load glucose concentration) with a high fasting glucose, results in the identification of a subgroup of people who are at high risk of progression to diabetes in a relatively short period of time [3]. Such a strategy makes eminent sense in a trial since statistical power to demonstrate an effect is driven, in part, by the absolute risk of the endpoint in question in the study population. However, this process of risk-group identification, which works so well in trials, is difficult to translate into real-world settings in which it is difficult to get OGTTs undertaken for diagnosis of diabetes, let alone for the purpose of risk-group identification. Thus, many implementation strategies for diabetes prevention in the real-world setting involve more practical ways of identifying high-risk individuals [11–13].

Another key distinction between diabetes prevention trials and the real world is that in a trial the focus is entirely on individuals without diabetes who are at high risk. People who are found by a measurement of glycaemia to have prevalent, but previously undiagnosed, diabetes are excluded. In a research paradigm this latter group of individuals are the principal focus of a different theme of research into the benefits of screening for diabetes. In real-world settings, screening for undiagnosed diabetes and the identification of people at high risk for prevention interventions cannot be seen as separate issues because they are inextricably linked by the processes of risk prediction and measurement of glycaemia. In turn, these two processes are linked to the more fundamental issue of how type 2 diabetes is defined and how it is diagnosed. The international move to accept the very easy to measure HbA_1c_ test [14] as a way of diagnosing diabetes has opened up more easily implementable ways of testing for prevalent but undiagnosed diabetes and identifying people with prediabetes who can be offered preventive interventions. This linking of diabetes diagnosis, screening and high-risk-group identification is critical to implementation in real-world settings. How a risk assessment and high-risk intervention programme could be implemented ought to be considered right at the outset of its design, not as an after-thought once it has been shown to work. This is an important principle that influences how we might think of future developments in diabetes prevention, including more personalised approaches.

## The relevance of the evolution of diabetes prevention interventions for personalised prevention

The brief résumé of how the field of diabetes prevention has evolved from the early trials to the current implementation of integrated diagnostic screening and high-risk prevention programmes serves a key purpose of showing where the field is currently. The development and evaluation of personalised approaches to diabetes prevention are not happening in a vacuum but in a context in which many countries are implementing more standard approaches and others are yet to even make that move. Where healthcare systems have already implemented diabetes prevention measures, the key question is whether more personalised approaches can augment the existing approaches. Without a focus on augmentation rather than replacement, there is a risk that a call for greater personalisation could undermine the implementation of approaches that demonstrably work in trials and for which considerable attention has been placed on the challenges of translation into real-world settings. Undermining an approach that is effective, admittedly only in part for most people, risks letting a drive towards the best be the enemy of the implementation of the merely very good. In healthcare systems where diabetes prevention programmes are yet to be implemented, personalised prevention approaches must be considered alongside the broader question of why diabetes prevention interventions that are known to be efficacious are not being implemented. There is no single reason for this as policy decisions are context specific. However, in less-well-resourced countries, the prospect of implementing integrated screening and high-risk diabetes prevention approaches is challenging when the strengthening of healthcare systems to deal with those with diagnosed disease is such a pressing concern [15]. In such settings, the consideration of greater personalisation of diabetes prevention is unlikely to be a priority.

## The extent to which current diabetes prevention approaches are already personalised

Personalisation of prevention approaches is a question of degree, not a binary alternative to non-personalised interventions. Existing approaches are already personalised to some extent because testing for glycaemia is not universal but is usually undertaken on the basis of an estimation of risk either by strategies that offer testing to particular population subgroups (e.g. people of a particular age or level of obesity or women with previous gestational diabetes) or following the quantification of risk at the individual level using risk predictors that include personal characteristics. Such prediction, using tools that utilise existing information with or without additional simple clinical variables, is already very good [16], creating a challenge for more personalised approaches to risk prediction. Given the principle already articulated in this article that personalised approaches need to augment rather than replace existing approaches, it follows that personalised prediction methods need to be evaluated against the extent to which they outperform existing approaches, not whether they work in isolation. Since existing approaches are already very good, this is a challenging proposition as the demonstration of enhanced prediction becomes incrementally harder as tools improve.

Perhaps the area where personalisation of prevention has the most to deliver is in the nature of the intervention. In established prevention programmes that build on the classical RCTs, the intervention is usually common to a group of people who are defined as being at risk by virtue of having prediabetes. While trial evidence does suggest that a focus of such interventions on weight reduction, increased physical activity and dietary change is logical on average for people who are identified as being in the target population [17], it is also true that there is heterogeneity among that target population. As an example, in early data from the National Health Service Diabetes Prevention Programme roll out in England [18], 44% of those referred to the programme with a diagnosis of prediabetes were obese and a further 33% were overweight. However, 15% of people had a BMI under 25 kg/m^2^ when referred. For this sizeable subgroup, the face validity of an intervention with a heavy emphasis on weight loss has some challenges from a participant perspective. It is true that in the US DPP the analysis of efficacy by population subgroups suggested that the lifestyle intervention was actually marginally more effective in those with lower BMI at baseline (RR reduction 65%) than in those with obesity (RR reduction 61%). Overall, there was no statistically significant heterogeneity by initial BMI [3], although the lower comparison group in this context comprised people with BMI 22–30 kg/m^2^ and were a mixed group of people, some of whom were overweight and some who were of normal weight. Whether the intervention was effective in those with BMI <25 kg/m^2^ was not reported. Similarly, within the Finnish Diabetes Prevention study there was no evidence of heterogeneity of the intervention effect by initial BMI. However, this trial was smaller than the US DPP and likely to be underpowered to detect subgroup effects, particularly when multiple potential interactions were tested [19].

There is clear evidence of heterogeneity of effect for pharmacological approaches to the prevention of type 2 diabetes, with the DPP trial showing that the RR reduction for metformin therapy in people with initial BMI >35 kg/m^2^ was significantly greater (at 53%) than in those with lower BMI [3]. In the subgroup of people with initial BMI 22 kg/m^2^ to <30 kg/m^2^, there was no evidence that metformin was effective in reducing the risk of progression to diabetes (risk reduction 3% [95% CI −36, 30]). This observation opens up the possibility of personalisation of pharmacological diabetes prevention on the basis of BMI. However, it is likely that such heterogeneity is specific to the mechanism of action of particular glucose-lowering treatments. While there is demonstrable BMI-dependent effectiveness for metformin, there is, for example, no evidence of heterogeneity of effect by initial BMI for pioglitazone [20].

It is well established that ethnicity influences individual risk of type 2 diabetes and that South Asian populations in particular develop type 2 diabetes at an earlier age [21] and at a lower level of overall obesity than people of Europid origin [22]. Ethnicity is thus one of the key factors that contribute to individual-level risk prediction or, if stratified approaches to prevention are considered, is a key factor defining population subgroups of interest on the basis of age, BMI and ethnic origin. However, evidence for differential responses to lifestyle interventions by ethnicity in classical trials is limited. Even in the largest single trial, the US DPP, which oversampled populations of non-white origin (representing 45% of the participants), there was no statistically significant heterogeneity of the intervention effect by ethnic group [3]. The authors of that classic paper were at pains to point out that ‘the study had inadequate power to assess the significance of effects within the subgroups’ and thus the failure to observe any demonstrable heterogeneity of effect by ethnicity or age, BMI and sex, cannot be taken as definitive evidence of the absence of true heterogeneity.

## Development of more personalised approaches to type 2 diabetes prevention

### Personalisation on the basis of individual risk

For a given level of effectiveness, individuals stand to gain more from preventive interventions if they are at high absolute risk. This is because the number needed to treat for any preventive or therapeutic intervention is the inverse of the absolute risk reduction. Therefore, if the relative effect of the intervention is the same in all individuals (an assumption that at least for classical interventions holds, as the previous section of this review describes), the number needed to treat is lower in people whose absolute risk is higher. For most people, the number needed to treat is a relevant and intuitive parameter when considering whether to engage with an individually targeted preventive intervention because it addresses the question of ‘how many people like me would need to participate in the intervention for one to avoid developing diabetes’. More personalised ways of predicting risk do, therefore, at least in theory, have the potential to contribute to personalised prevention if they more accurately identify those at high absolute risk.

The past 15 years has seen a considerable expansion in the number of genetic loci definitely known to be associated with type 2 diabetes, as a consequence of the combination of technological innovation in genotyping at scale, the application of these methods in large-scale studies and previously unprecedented levels of international collaboration and result sharing [23]. These studies have provided important insights into the aetiology and pathophysiology of type 2 diabetes but their role in risk prediction has been more limited. Early studies, such as the Framingham Study, using a genetic risk score (GRS) based on 18 single nucleotide polymorphisms (SNPs) to predict the future development of diabetes, showed that the genetic score made a non-significant marginal difference to the area under the receiver operating characteristic curve (aROC; 0.900 without and 0.901 with the genotype score) [24]. This observation underscores the point made previously in this review that existing methods for predicting type 2 diabetes based on simple clinical and biochemical variables are extremely good. Of course, it is possible that better genetic risk prediction could be obtained using updated evidence of association with type 2 diabetes from genome-wide association studies to create a more extensive GRS, or simply by using the association of all genetic variants across the genome in a polygenic risk score. However, most of the predictive utility of a genetic score comes from the relatively small group of strongly associated alleles and the marginal advantage of adding large numbers of more weakly associated loci is limited. An update of the Framingham analysis in 2014 used a GRS based on 62 SNPs and showed virtually identical results to the earlier analysis, with an aROC of 0.903 without the genotype score and a non-statistically different aROC of 0.906 with it [25]. Extending analyses to the whole genome adds very little additional predictive utility. In an analysis based on data reported in Mahajan et al [23], Udler showed that a GRS with only age and sex as additional covariates had an aROC of 0.72, whereas one using a polygenic risk score across the whole genome had an aROC of 0.73 [26].

Even if genetics adds little to prediction over existing risk scores for the whole population, it remains possible that it might enhance prediction in population subgroups, if there is an interaction between a particular characteristic and genetic risk. The examination of interaction between exposures and incidence of type 2 diabetes is challenging since it requires very large studies with standardised assessment of key exposures and long follow-up. In the EPIC-InterAct study, 12,403 incident cases of type 2 diabetes were ascertained in a large population-based cohort study originally involving centres from eight different European countries with nearly 4 million person-years of follow-up [27]. A GRS based on 49 loci was strongly associated with the incidence of type 2 diabetes (HR per SD of the GRS was 1.41 [95% CI 1.34, 1.49]) [28]. On a relative scale there was evidence of interaction between this GRS and BMI at baseline, with the HR per SD of the GRS being greatest in those who were thinnest at baseline. However, it is the absolute (rather than relative) risk that is most relevant to prediction and prevention and, on an absolute scale, the marginal change in risk linked to differences in underlying genetic risk was dwarfed by that of the non-genetic risk factors, particularly obesity.

Finally, it remains possible that genetic factors for type 2 diabetes could influence the response to a preventive intervention. The challenge of demonstrating this is probably comparable to that of testing for interaction in observational studies, but the sample sizes available for analysis are much smaller. Even in the largest prevention trial, the DPP, there was no evidence of a difference in the effect of lifestyle by genetic risk of type 2 diabetes as assessed by a 34 SNP GRS (*p* for interaction=0.13) [29]. Because of the limited statistical power to examine heterogeneity of intervention effect by subgroups, the possibility of difference in response to intervention by genetic risk groups remains an unanswered question.

Technological advances in measurement of epigenetic markers, metabolites and proteins have made it possible to add other ‘omic’ measures to risk tools for prediction of type 2 diabetes. As with the genetic markers, the available evidence suggests that each of these types of additional ‘omic’ data provide novel insights into disease aetiology and pathogenesis [30–32] and although they are predictive of disease, especially when considered in isolation, they do not make a material difference to the predictive utility of risk tools when considered as an addition to existing information [33]. The next step forward is not to keep repeating the same mistake of hoping that the addition of yet further information will somehow improve the prediction of type 2 diabetes but instead to reflect on the principle that diagnosis, screening and high-risk prevention are part of the same process, and to consider whether personalisation of prevention may play a role in specific diagnostic subgroups that are hidden within the diffuse disorder that we label as type 2 diabetes.

### Personalisation of prevention by breaking type 2 diabetes down into diagnostic subgroups

It is generally accepted that type 2 diabetes is a heterogeneous disorder and that efforts to break it down into specific subgroups could have utility for personalised treatment and potentially for prevention. For example, Ahlqvist et al used a data-driven approach to define five subtypes of diabetes using six key variables: GAD antibodies; age at diagnosis; BMI; HbA_1c_; and HOMA assessments of beta cell function and insulin resistance [34]. This analysis clearly identified a subgroup of individuals with autoimmune diabetes characterised by early onset disease, relatively low BMI, insulin deficiency and GAD antibody positivity. Putting that group to one side, the remaining individuals could be put into four further subgroups: a severe-insulin-deficiency subgroup; a severe insulin resistance subgroup; an obese but not insulin-resistant subgroup; and a subgroup termed ‘mild age-related diabetes’. Although these subgroups are associated with different patterns of diabetes complications and may be associated with response to therapy, the value of clusters in predicting response was less than for predictions based on simple clinical features, raising doubts about the value of these clusters for personalised therapy [35]. The clinical utility of the clusters for personalised prevention is even more challenging since many of the features that define the clusters are not knowable at the time preventive interventions would be applied, since they are characteristics that are manifest at the time of diagnosis. This would not be a limitation if the clusters were linked to clearly distinct aetiological pathways that could be detected prior to diagnosis. In their original paper, Ahlqvist et al showed that, although there was heterogeneity in the association of established type 2 diabetes genetic loci with the five disease clusters, the magnitude of the differences in association were small and there were no loci that were uniquely associated with individual clusters. GAD antibody positivity at the time of diagnosis is part of the Ahlqvist subgrouping. It is also associated with risk of future diabetes if measured in people without diabetes [36], but the level of association is modest which, with the low frequency of antibody positivity, makes the predictive value limited. Even if the autoimmune subgroup was identifiable at an earlier stage, the nature of the preventive intervention that would be put in place is uncertain. The form of clustering used by Ahlqvist et al essentially forced individuals to be members of only one cluster. An alternative approach was used by Wesolowska-Andersen et al, who used a soft-clustering method that allowed individuals to be members of multiple clusters linked to five aetiological processes: insulin secretion; obesity; insulin resistance; dyslipidaemia; and reduced beta cell glucose sensitivity [37]. The utility of these soft clusters for personalised treatment or prevention is unclear. A key observation in this study was that most individuals with newly diagnosed diabetes belonged to multiple clusters, suggesting that a mixed aetiology for type 2 diabetes is the norm rather than the exception.

An alternative approach towards breaking down all cases of type 2 diabetes into subgroups is to tackle heterogeneity by identifying specific diagnostic subtypes at the margins that can be distinguished as having a specific cause. This fits with the reality of type 2 diabetes, which is in effect a diagnosis of exclusion since it has no simple operational definition and is often defined by what it is not (i.e. it is a diagnostic label for a type of diabetes that is not type 1 and for which there is no specific known cause). This perspective in itself certainly has clinical value as it ensures that clinical attention is kept on the possibility of specific causes, recognising that diagnostic labels like ‘type 2’ can get in the way of appropriate diagnosis and treatment if applied blindly without consideration of alternative specific causes. In the context of a discussion about personalised prevention, the possibility that there are some covert specific subgroups of diabetes hidden within the population of people with type 2 diabetes raises the possibility that the identification of that subgroup might identify opportunities for personalised prevention, particularly if the aetiological process linked to that subgroup were distinct from that of more typical type 2 diabetes. One key example of this could be familial partial lipodystrophy, which not only exists as a distinct phenotype with particular genetic causes but is also probably covertly present in the population of people labelled as having type 2 diabetes but distinguishable on the basis of being enriched with genetic loci linked to insulin resistance [38]. In this instance, separation of a phenotypic subgroup could have advantages both for more personalised treatment and prevention (e.g. through specific therapies targeting the primary pathophysiological defects in adipose tissue). What distinguishes this example from the more blanket approach of applying GRSs to personalised prevention of type 2 diabetes is not only the specificity of the phenotype but also the potential application of a preventive intervention distinct from that offered to the general population of those at high risk.

### Personalisation of prevention by greater individualisation of the targets for preventive action

It would be perfectly possible for preventive intervention programmes to be much more adaptive so that they were tailored not only to the specific behaviours that an individual needed to change (rather than simply a standard set of risk factors) but also, perhaps more importantly, to those risk factors that the individual was willing and able to prioritise for change. Additionally, it would be possible for interventions not only to provide broad generic recommendations about behaviour change but also to provide assistance or prompts for behaviour change at certain times when it might be particularly effective, such as around critical points when decisions between alternatives choices are being made.

A complementary approach to trying to distinguish personalised prevention by greater specificity of pathophysiological pathways to diagnostic subgroups of type 2 diabetes is to focus more on the potential for greater individualisation of the behavioural risk factors that drive the disease. The promise of this personalised nutrition approach is considerable but the science is in its infancy. In an important paper in 2015, Zeevi and colleagues showed that a machine learning algorithm bringing data together from diverse sources, including blood biomarkers, diets, anthropometry, physical activity and the gut microbiome, could predict individual glycaemic responses to meals in free-living individuals [39]. Importantly, they also undertook a small RCT in which 28 individuals were randomly assigned to receive dietary recommendations from a clinical dietitian based on their expert interpretation of postprandial glucose response to meals during a profiling week or to receive dietary advice based on the machine learning algorithm. In both instances, participants were provided with a dietary recommendation for 1 week that was likely to be beneficial in relation to postprandial glucose responses and one that was likely to be disadvantageous. The trial showed that some of the postprandial glucose response variables were lower during the week in which participants consumed the predicted beneficial diet, although there was no major difference between whether that prediction came from an expert or from the machine learning algorithm. How such personalised nutrition recommendations would impact on clinical outcomes over the long term was not investigated in this initial study. Nor were they investigated in similar work on machine learning algorithms that predict individual glycaemic responses to food intake conducted in the Predict-1 study [40].

However, in a follow-up trial in 225 people with prediabetes, the Segal group undertook an RCT of what they termed a personalised postprandial targeting (PPT) diet, which they compared with a group assigned to follow a Mediterranean diet in a 6 month dietary intervention period with a 6 month post-intervention follow-up [41]. As in the previous study, the PPT diet was tailored to individual participants based on their postprandial glucose responses using a machine learning algorithm. There were three primary outcomes in the trial: the total time during the day when glucose measured by the continuous glucose monitor was above 7.8 mmol/l; HbA_1c_; and an OGTT undertaken at home. There was a higher loss to follow-up rate in the PPT arm, explained by the authors as being related to higher motivation of participants in the group assigned to the Mediterranean diet, who were promised personalised predictions from the algorithm at the end of the follow-up period. Of course, such an observation could also be explained by lower motivation to continue in the PPT group for whatever reason. The between-randomised-group difference in differences (between baseline and follow-up) were statistically significant for two of the primary outcomes but not for the OGTT. Importantly, the magnitude of effect seen in the PPT group, with a difference of 0.18% in HbA_1c_ between baseline and follow-up, was comparable with that reported in the DPP [3].

A key question that arises from this study concerns the explanation for the differences between the dietary intervention groups. Although weight loss was greater in the PPT group, the difference from the group randomised to the Mediterranean diet was not statistically significant. Nor was there a difference in physical activity between groups. However, the PPT intervention resulted in a diet that was significantly lower in carbohydrates. At 6 months, the average percentage of carbohydrate intake in the Mediterranean diet group was 42.4% of total energy, which was double that of the PPT group (20.4%). This raises the question about whether the comparison being tested here is between a low- and a high-carbohydrate diet rather than anything to do with personalisation of the dietary recommendation per se.

A characteristic of this field is the rapidity with which techniques for personalised prediction become commercialised, with both of the approaches mentioned above already being available from companies linked to the scientific groups that undertook the original research: DayTwo [42]; and Zoe [43]. Whether the promise of such approaches is matched by evidence of their long-term beneficial impact remains uncertain but interest and activity in this field is growing rapidly, as evidenced by the award of $170 million by the US National Institutes of Health for a new study of 10,000 people which will develop algorithms to predict individual responses to food and dietary routines [44]. Even with large traditional funder investment, this field will develop rapidly in the commercial sector, with large providers positioning themselves to offer ‘innovative tools, personalised data and insights, and an ever-growing library of programs designed to help’ paying clients discover what works for them [45]. While this commercialisation of personalised prevention is not itself a bad thing, it does raise considerable challenges for the future, particularly in relation to knowing what is in the ‘black box’ of proprietary interventions that change with time and may not be subject to the same sort of rigorous evaluative assessment as more traditional preventive interventions. In the end, since such interventions are neither being offered by state providers nor recommended by them, whether an individual wishes to use them is a matter of individual choice and since this is a financial transaction, the principle of caveat emptor applies. Whether regulators such as the US Food and Drug Administration (FDA) or the UK Medicines and Healthcare products Regulatory Agency (MHRA) regulate the claims that are made about such personalised prevention approaches depends upon the intended purpose and consequently on decisions about whether such approaches fall under medical device regulations [46].

### Personalised and whole-population approaches to prevention

A final key question is where increasing efforts to personalise prevention of type 2 diabetes leaves interventions that take a whole-population approach. From this perspective, diabetes is seen less as a clinical presentation of a pathophysiological defect but rather as a public health manifestation of a societal problem. Unhealthy diets and low levels of physical activity can be viewed not solely as the consequences of individual choices but as the product of broader issues such as the food system and the built environment. Preventive interventions that influence those broader issues have the potential to influence behavioural risk factors in the whole population and small changes in a large number of individuals can have a sizeable population-level impact. In the past some have attempted to argue for greater investment in research into whole-population prevention by framing this as an alternative to more individualised approaches [47]. In reality this is a false dichotomy and it is perfectly rational for a prevention strategy in any given country to include both approaches, although the balance of investment aimed at different approaches to prevention depends on various factors including the capacity of the healthcare system.

Whatever the future holds for more personalised approaches to type 2 diabetes prevention, it is important that the investment and effort that is put into such developments at best synergises with, but at the very least does not detract attention from, either the roll out of established individual-level interventions or the implementation and evaluation of societal-level prevention strategies.

## Abbreviations

aROC: Area under the receiver operating characteristic curve
DPP: Diabetes Prevention Program (US trial)
GRS: Genetic risk score
IGT: Impaired glucose tolerance
PPT: Personalised postprandial targeting
